# Editorial: Immune Response to Biofilms

**DOI:** 10.3389/fimmu.2021.696356

**Published:** 2021-06-07

**Authors:** Giovanna Batoni, Luisa Martinez-Pomares, Semih Esin

**Affiliations:** ^1^ Department of Translational Research and New Technologies in Medicine and Surgery, University of Pisa, Pisa, Italy; ^2^ School of Life Sciences, University of Nottingham, Nottingham, United Kingdom

**Keywords:** biofilms, biofilm-associated infections, innate immunity, adaptive immunity, immune response

Biofilms are one of the most widely distributed and successful form of microbial life and are associated to a significant amount of human infections (1). They typically contain aggregates of microorganisms adhering to a substrate and embedded in a self-produced matrix of extracellular polymeric substances. Importantly, biofilm-associated microorganisms exhibit an altered phenotype with respect to growth rate and gene transcription that provide them with unique characteristics as compared to their planktonic counterparts (2). These include the ability to resist antimicrobial treatments and host immune responses rendering biofilm-associated infections one of the major threats of the modern medicine. Despite the recognized clinical importance of biofilms, the vast majority of studies of the immune response against pathogens focuses on microorganisms in the planktonic state whereas the immune response against infectious biofilms has been far less investigated. There is evidence that the host immune response is only partially beneficial in clearing biofilm-associated infections if not even harmful by accelerating collateral tissue damage, as is seen in *Pseudomonas aeruginosa* biofilm-associated lung infections in Cystic Fibrosis (CF) patients (3). Therefore, it is critical to understand the complex interactions that establish between biofilms and the immune system as this may help in identifying new targets and strategies of immune intervention against biofilm-associated infections. We hope that this Research Topic may contribute to this purpose by collecting a number of papers (9 articles from 60 authors), exploring different aspects of the immune response to microbial biofilms.

The study of the immune response to biofilms is highly dependent on the development of appropriate *in vitro* and *in vivo* experimental models allowing to realistically figuring biofilm-immune cell interactions. In this respect, the Research Topic includes at least three articles. In the first one, Kaya et al., established an *in vitro* host cell-biofilm interaction model suitable to investigate the human peripheral blood mononuclear cell response (PBMC) to *P. aeruginosa* biofilms. Interestingly, the results obtained demonstrated that not only *P. aeruginosa* biofilms induced marked activation and response of PBMC, but also that PBMC or their supernatants caused a significant increase in biofilm-associated *P. aeruginosa*, suggesting a reciprocal complex interaction between host blood cells and the bacterium. A novel murine model of *Staphylococcus aureus* implant-associated infection was developed by Gries et al. Of note, the authors applied for the first time time-lapse intravital multiphoton microscopy to simultaneously visualize in real-time *S. aureus* biofilm formation and immune cell activity. Using this innovative approach, they demonstrated that *S. aureus* biofilms impede neutrophil chemotaxis, redirecting their migration patterns to prevent biofilm invasion. Finally, a RNA sequencing-based approach was used by Heravi et al., to depict the whole transcriptomic profile in diabetic foot infection (DFI) tissues, contributing to clarify the role of the host inflammatory status in the progression of DFIs.

One of the most studied biofilm-forming bacteria, often taken as model organism in biofilm studies, is *P. aeruginosa*, a key pathogen in CF lung infections and chronic wounds (4). Moser et al. greatly contributed to this Research Topic with an updated and exhaustive review focused on the immune responses to *P. aeruginosa* biofilm infections. The mechanisms involved in the activation of the immune responses, the effector functions elicited by biofilms and their role in tissue damage, as well as the mechanisms by which the biofilms evade immune responses, and potential treatment strategies are discussed in detail in the review.

A number of articles of the Research Topic addresses the inhibitory and/or dysregulating effects of microbial biofilms on the innate immune responses. For instance, in their article, Kernien et al. report that neutrophils collected from patients with invasive candidiasis as well as from healthy donors fail to release neutrophil extracellular traps (NETs) in response to *Candida albicans* biofilms. As NETs exert antifungal activity (5), inhibition of NETs release by *C. albicans* biofilms may very well represent a mechanisms of immune evasion likely contributing to the resilient nature of *Candida* biofilm infections of medical devices (6). The existing knowledge on the role of biofilm-innate immune interactions in driving immune dysregulation and persistent inflammation in chronic wounds was summarized in the comprehensive review of Versey et al. that also illustrates novel treatments currently under development to target these interactions. The article by Miller et al. points out the possible role played by biofilm-products in the genesis or exacerbation of a number of inflammatory human disorders. In particular, the article focuses on amyloid curli, secreted by Gram-negative enteric bacteria, that makes up as much as 85% of the extracellular matrix of enteric biofilms. A thorough discussion on how amyloid-containing biofilms may act as triggers of inflammation and self-assembly of pathological human amyloids, contributing to the pathogenesis of gastrointestinal, autoimmune, and neurodegenerative diseases is provided.

Other two interesting, but relatively poorly investigated aspects of the immune response to biofilms are dealt in the articles of De Morais et al. and Trikha et al., respectively. In the first one, differences in the immune responses elicited by *S. aureus* biofilms in the central nervous system (craniotomy-associated infections) as compared to biofilm infections in the periphery are highlighted, emphasizing the critical role of niche-specific factors in driving *S. aureus* biofilm-leukocyte crosstalk. In the second paper, Trikha et al. demonstrated that angiotensin-converting enzyme inhibitors (ACEi), often utilized for treating hypertension, increases *S. aureus* burden and impairs immune responses in a preclinical model of implant-associated infections, raising the intriguing issue that commonly used drugs may negatively impact the immune response to microbial biofilms.

Overall, we believe that the articles collected in this Research Topic represent a step forward for a better understanding of the host immune response to microbial biofilms and hope that they may stimulate further studies in this interesting research field. Such studies could pave the way for the development of new preventive and/or immune-therapeutic approaches able to dampen the harmful activities of the immune system, meanwhile activating the branches of the immune system that can eradicate biofilm-infections without causing detrimental collateral damage.

## Author Contributions

All authors contributed to the article and approved the submitted version.

## Funding

This work was supported by institutional funds from the University of Pisa.

## Conflict of Interest

The authors declare that the research was conducted in the absence of any commercial or financial relationships that could be construed as a potential conflict of interest.
